# Application of Laser-Induced Breakdown Spectroscopy and Chemometrics for the Quality Evaluation of Foods with Medicinal Properties: A Review

**DOI:** 10.3390/foods11142051

**Published:** 2022-07-11

**Authors:** Muhammad Hilal Kabir, Mahamed Lamine Guindo, Rongqin Chen, Alireza Sanaeifar, Fei Liu

**Affiliations:** 1 College of Biosystems Engineering and Food Science, Zhejiang University, 866 Yuhangtang Road, Hangzhou 310058, China; mhkabir@atbu.edu.ng (M.H.K.); guindo@zju.edu.cn (M.L.G.); chenrq@zju.edu.cn (R.C.); asanaei@zju.edu.cn (A.S.); 2 Department of Agricultural and Bio-Resource Engineering, Abubakar Tafawa Balewa University, Bauchi 740272, Nigeria; 3 Key Laboratory of Spectroscopy Sensing, Ministry of Agriculture and Rural Affairs, Hangzhou 310058, China

**Keywords:** laser-induced breakdown spectroscopy, plasma, spectroscopy, medicinal properties, chemometrics, quality

## Abstract

Laser-induced Breakdown Spectroscopy (LIBS) is becoming an increasingly popular analytical technique for characterizing and identifying various products; its multi-element analysis, fast response, remote sensing, and sample preparation is minimal or nonexistent, and low running costs can significantly accelerate the analysis of foods with medicinal properties (FMPs). A comprehensive overview of recent advances in LIBS is presented, along with its future trends, viewpoints, and challenges. Besides reviewing its applications in both FMPs, it is intended to provide a concise description of the use of LIBS and chemometrics for the detection of FMPs, rather than a detailed description of the fundamentals of the technique, which others have already discussed. Finally, LIBS, like conventional approaches, has some limitations. However, it is a promising technique that may be employed as a routine analysis technique for FMPs when utilized effectively.

## 1. Introduction

Laser-induced Breakdown Spectroscopy (LIBS) has become increasingly popular in recent years due to its distinctive features, including its broad application, minimal or no sample preparation procedures, speed, affordability, and the ability to sense remotely in a variety of scientific fields and applications [1]. LIBS analyzes the presence of spectral emissions from laser-induced plasmas using optical emission techniques. This technology was developed in the 1960s. LIBS applications have become popular due to their advantages over conventional methods. The development of Q-switched pulse lasers in 1963 is considered the precursor to LIBS. Nd: YAG lasers are the most commonly used for LIBS applications since they produce highly focused pulses of high energy. Over the years, LIBS has gained attention for its potential in many fields.

A multi-element analytical technique is successfully employed in various disciplines, including space exploration, metallurgy, forensics, and pharmaceuticals [2]. Since then, researchers have become increasingly intrigued by LIBS technology for having great potential in many fields. It allows for both qualitative and quantitative analysis of trace elements using LIBS. Several trace elements from medicinal plants with hypoglycemic effects were studied using this technique in 2008 [3]. It consists of measuring the intensity of spectral emissions from laser-induced plasmas. Specifically, the intensity of plasma emission is directly proportional to the abundance of an element. It is an excellent method for analyzing various materials and compounds due to its relative simplicity and ability to analyze solids, liquids, or gases at the same time. However, quantitative evaluation of the elemental compositions determined using LIBS measurements is more difficult than purely numerical analysis. In recent years, this technology has been utilized in analyzing environmental and biological samples, advanced materials, such as semiconductors, analyses of online samples, remote analyses of nuclear power plants, and depth profiling [3]. Researchers are currently studying a LIBS-based methods for the non-invasive and rapid examination of foods with medicinal properties (FMPs). The system is fundamentally optical. Chemical compositions can be analyzed qualitatively and quantitatively using this technique. It provides process analytical technology (PAT) and handheld functionality [4,5,6,7,8,9,10,11]. There is, however, an increasing interest in developing new analytical instruments and approaches to increase the sensitivity of LIBS, reduce the effect of sample matrix on LIBS, and test both well-established and novel chemometric approaches [12]. Some recent articles have discussed different methods and techniques for analyzing FMPs. However, few articles provide a comprehensive review of the applications of LIBS in FMPs detection. A comprehensive overview of recent advances in LIBS is presented, along with its future trends, viewpoints, and challenges. Besides reviewing its applications in both FMPs, it is intended to provide a concise description of the use of LIBS and chemometrics for detecting FMPs, rather than a detailed description of the fundamentals of the technique, which others have already discussed.

## 2. Principles Related to LIBS

**Figure 1:**
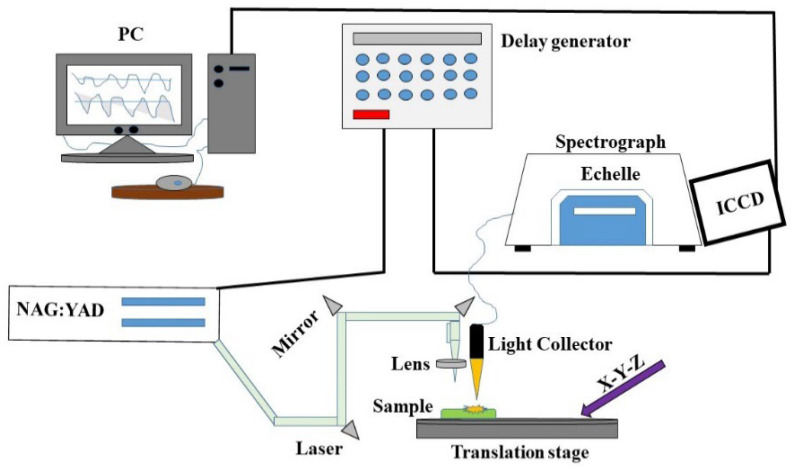
Schematic diagram of the LIBS setup.

**Figure 2:**
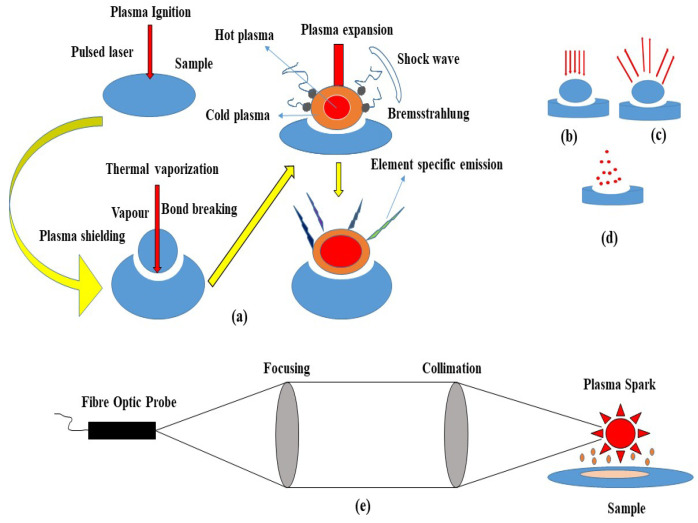
(**a**) Schematic of the Laser-Induced Breakdown Process. (**b**) Plasma ignition. (**c**) Plasma expansion and cooling. (**d**) Particle ejection and condensation. (**e**) An example of a light acquisition system.

Typically, LIBS systems consist of a few components that can be configured to meet specific requirements for scientific research, or they can be compact and rugged for field measurements in the field. Generally, these can be divided into (i) short solid-state pulses, (ii) Q-switched lasers, (iii) optics, (iv) detectors and spectrometers, and (v) computer systems. This system can provide a close-up analysis and a standalone configuration (Figure 1). In LIBS, a pulsed laser is used at high power on a sample surface (1), which causes the molecules of the sample to separate into their constituent atoms. A small amount of the sample is converted into vapor as a result of rapid energy discharge (2), resulting in the formation of high-temperature micro plasma accompanied by the characteristic sound of an ultrasonic shockwave (3) [13]. The plasma loses its energy as soon as it expands into space and begins to atomize. A spectrometer can detect the light emitted by ions and atoms during the relaxing stage. The sample is subjected to a laser pulse with a high peak power to cause a spark breakdown in the test sample medium. When a laser pulse is applied, the plasma reaches temperatures of up to 4000–15,000 K [13]. Molecules and particles within the sample are dissociated due to the energetic spark. Electrons and ions thereby excite the atoms. Cooling the plasma allows the excited ions and electrons to return to their ground states. Each emits wavelengths specific to its element and its fingerprint (signature). The composition can be determined by measuring the wavelengths and intensities of specific atomic emission lines (Figure 2).

Time-resolved LIBS measurements are the most common LIBS measurement used for improving signal-to-background ratios and minimizing the amount of continuum background generated during the initial plasma phase. Theoretically, plasma emission lines are analyzed under local thermodynamic equilibrium (LTE) conditions. The LTE can determine the plasma temperature and electron density, which can then be used to study ionization and atomization processes. Laser-induced plasma can vary significantly in size and shape depending on the ambient conditions, such as pressure, gas composition, and gas density [14]. Machine learning (ML) is considered a technique for learning from enormous amounts of data and extracting the unknown features for estimation or labeling. Typically, sample data are analyzed to determine whether there is a relationship between input and output data. It has generally been applied in emerging areas, especially in determining the authenticity of herbs. The complexity and diversity of the herb complicate LIBS spectral analysis, as well as a large number of factors. LIBS can extract the maximum amount of relevant data combined with machine learning techniques. This allows for greater accuracy in classification and quantitative analysis. Despite the volatility of sample spectra, LIBS coupled with machine learning can frequently offset this problem to a great extent. In recent years, ML has made significant advances beyond the traditional chemometric method [15]. It now provides robust and continuously updated algorithms and tools for developing spectroscopic data treatment models [16,17]. RF, for example [18], SVM [19], etc., have been utilized widely in quantitative and qualitative LIBS spectral analyses. Leo Breiman introduced RF in 2001, a regression technique based on multiple classifiers [18]. This strategy is based on the bootstrap method, which is applied continuously in creating test and training samples. RF generates several classification tree forms, and final prediction results are based on a simple majority vote of a single classification tree. In addition to being a useful technique for prediction, RF can also provide higher levels of accuracy. However, these algorithms suffer from a number of inherent shortcomings, which, in turn, has restricted their use in LIBS. Most traditional neural networks use gradient descent algorithms to update their parameters. Thus, the training process is slow, and there is a risk of falling into a local optimum. Thus, many new algorithms have been developed that are highly efficient in generalization at very fast learning rates to overcome these disadvantages. Kernel-based extreme learning machine (K-ELM) is a relatively new nonlinear method that has demonstrated excellent performance in classification and regression applications. As a result, a kernel function can replace the hidden layer of ELM and be used to eliminate the problems presented by traditional neural network training algorithms in terms of speed and over-fitting [20].

### LIBS Plasma Production

In plasma production, pulsed lasers are commonly used (LIBS). Specifically, laser characteristics are crucial to plasma production in gaseous, liquid, and solid media. Short-duration laser pulses with different wavelengths can generate multi-million-watt laser pulses from the infrared to the ultraviolet spectrum. Advanced lasers can produce vibrations that contain many billions to trillions of watts. In a fraction of a second, high-power laser pulses can vaporize metallic or refractory surfaces. Powerful lasers capable of delivering their energy to specific locations are significant. More importance is placed on the power per unit area provided to the target rather than its absolute value in LIBS. Depending on the laser wavelength, a power unit is called an “irradiance.” Conventional light sources with kilowatts of power are unable to focus as well as laser radiation and, therefore, cannot produce the same effects that lasers can [21].

## 3. The Theoretical Approach of LIBS

Understanding the principles of plasma physics to provide an optimal environment for LIBS measurements is essential. This atomic-level chemical analysis technique is influenced by several factors, including the environment and climate [22] In practice, LIBS is an easy method to implement. The creation of plasma is accomplished by a laser beam (typically pulsed infrared radiation of 1.06 mm wavelength) through a lens. The data are transmitted via fiber optic cable, and the collected light is sent to a spectrometer for analysis [23]. Much progress has been made in studying plasma generation physics in recent years. The plasma of LIBS is characterized by local thermodynamic equilibrium (LTE) models, hydrodynamic and kinetic models, non-uniform plasmas, and plasmas generated in a vacuum [23]. Plasma comprises atoms, ions, and free electrons arranged locally in which the charged species cooperate. Several parameters relate to plasma classification, including the degree of ionization. A weakly ionized plasma has a ratio of electrons to other species of below 10 percent. A highly ionized plasma may lose atoms, resulting in very low electron to atom/ion ratios. LIBS plasmas are generally considered weakly ionized plasmas. LIBS exhibits a background continuum that decays more rapidly with time than the spectral line. The continuous oscillations are caused by bremsstrahlung (free–free) and recombination (free–bound). The process of bremsstrahlung produces photons by accelerating or decelerating electrons by collision. When an electron is free, it plunges into an ionic or atomic energy level and releases its excess energy as a photon. Based on the time resolution of plasma light, LIBS can distinguish between regions where signals of interest are dominant [24]. LIBS can be applied to solids and liquids and is generally an atomic emission spectrum analysis method for determining the composition of various materials [25].

Many factors contribute to the popularity of LIBS-type spectroscopy: it requires no sample preparation, it can be used for neutral, ion spectroscopy and stand-off measurements, and it is simple, inexpensive, compact, and portable. This distinctive quality has led to an increasing number of LIBS applications in recent years, permitting different experimental configurations tailored to specific application needs. A summary of the LIBS instrumental techniques examined in this chapter describes how the LIBS system of measurement operates and how its configurations and properties impact its measurements. It involves focusing laser pulses of short duration at the sample during analysis (typically lasting only a few nanoseconds or even a few femtoseconds). The energy impinging upon the matter in the vicinity of an irradiated area causes a localized area of above-normal temperatures and electron densities to manifest (a process known as a breakdown). Several factors can influence the plasma ignition process, including the physical properties of the excitation pulse (dimensions, such as wavelength, duration, intensity, repetition rate, etc.) and the material being irradiated. For plasma to form, some materials must be vaporized, and a small amount must expand at supersonic speeds perpendicular to the target’s surface. The plasma plume should contain the same composition as the target material to achieve stoichiometric ablation. Plasma emission of electromagnetic radiation can be analyzed to reveal its local spectral composition, which can be used to determine its elements. The delay at which the waveform is captured must be considered in the spectrum capture process. This spectrum appears as a combination of broad emission lines (mainly those associated with the Stark effect) superimposed over a continuous background resulting from both the transitions between bound and free electrons (bremsstrahlung) and electrons that are recombined with those that are bound (free to bound electron recombination). The continuous background intensity is rapidly reduced by ions capturing electrons within a few hundred nanoseconds. The atomic emission lines weakened and narrowed simultaneously due to bound electronic transitions. A delay of more than ten seconds leads to slow degradation of atomic lines, but simple emission signals begin to appear. For optimal thermodynamic equilibrium conditions, the duration of quantitative applications should not exceed a portion of the total duration of plasma emission [26].

Several LIBS-based instruments have been developed but are not widely used. The method is a potentially useful technique for various purposes in recent years. The significant technical advances in components, such as lasers, spectrographs, and detector lasers used in LIBS instruments, and the requirement to perform measurements under conditions for which conventional analytical methods cannot be used, have led to this change in technology. A literature review on LIBS indicates that the method can detect several elements comparable with or exceeding those of other field-deployable methods [26].

## 4. Advantages and Disadvantages of LIBS

LIBS can deliver an effective response within a short period, high-volume and real-time analytical results both in conventional laboratory settings and in the field. Compared to existing methods, many advantages improve its utility as an analytical technique for materials [27]. LIBS can detect all elements using a single laser pulse with a broadband spectrometer. Although LIBS must follow complex and time-consuming procedures, it does not require much sample preparation compared to other lab-based methods. Compared to many other currently available techniques, its instrumentation purchases and operating costs are less expensive. As it provides high lateral spatial resolution, particles can be examined individually using its real-time visualization of mineral grains or inclusions [28]. A LIBS analysis can be considered minimally destructive since each laser pulse uses only a few nanograms of material. A combination of LIBS and complementary methods enables simultaneous analysis of multiple elements in orthogonal orientation. A stand-off apparatus collected LIBS and RAMAN spectra from minerals of different types [29,30]. Techniques used in all analyses have their limitations. This holds for LIBS, and certain shortcomings should be considered when experimenting. LIBS experiments generally have lower limits of detection and precision than established methods. Despite this, they are often enough to distinguish between samples from different sources.

Due to the nanosecond laser pulses’ uneven energy distribution and differential coupling to the sample surface, the method suffers from matrix effects and shot-to-shot variability. Grain size, texture, reflectivity, and hardness may affect surface structure and physical matrix effects. Surface roughness affects the degree of laser energy coupling and LIBS signals of varying intensities [31]. In-homogeneity in a matrix can be controlled in several ways: homogenizing the sample (which negates a significant advantage of LIBS), implementing and selecting anomalous spectra by an algorithm that does not represent the entire sample interrogating it with laser pulses of hundreds or even thousands [32]. Chemical matrix issues arise when one element influences another element’s emission characteristics. If two different host materials contain the same component concentration, the LIBS emission intensity of the component will be different [33]. Thus, matrix-matched standards are difficult to find for LIBS measurements of biological samples. However, this phenomenon may impede the quantitative differentiation of a LIBS spectrum for a given instance, enhancing the qualitative differentiation of a LIBS spectrum. LIBS can perform internal or external quantitative analyses through calibration [34].

## 5. Prospects Related to Using LIBS in Herbal Technology

LIBS faces several challenges, primarily its acceptance in spectroscopic analysis. Although calibration-free algorithms are close to achieving this objective, the outcomes are not entirely accurate [13]. Some recent works improve the final result using spectral normalization [13] or a system that can automatically determine the elements of a sample [35]. LIBS may represent the most crucial goal, offering LIBS a place among the most popular spectrochemical techniques. For its widespread use in real applications, new advanced instruments must be developed that are cost-efficient and highly versatile. Obtaining an accurate analysis requires a cumbersome and expensive setup; the size and complexity of LIBS setups are currently being reduced. The recent development of micro LIBS and advancements in laser sources can profit LIBS research by applying the exact and concise setup in the field [13]. It was recently hyphenated with other spectrochemical techniques to combine their advantages. For instance, MSL is an excellent example of an integrated LIBS/Raman system [36,37]. LIBS analysis has recently taken on new forms following new techniques and approaches, including optical catapulting and molecular LIBS [36,37]. The laser pulse used in this procedure is below the plasma threshold energy; it creates solid aerosols that can be analyzed using LIBS. Another form of LIBS is molecular LIBS, which explores the molecules emitted from sample ablation and recombination between elements in the target solution and the surrounding atmosphere [13]. This capability can be added to LIBS to analyze organic samples [38].

Furthermore, improvements are needed in system stability, self-absorption, line broadening, and high intensity of the background continuum, as well as the strong matrix effect. The development of LIBS instrumentation progresses very rapidly, so it appears likely that these improvements will likely overcome most of the drawbacks above. Additionally, chemometrics and data evaluation will enhance the reliability of the results. LIBS has yet to be established as a valid analytical technique for herb analysis, and there are significant research needs both in the research field and at the production level.

## 6. Quality Assurance, Chemical Constituent and Risk Assessment of Foods with Medicinal Properties Using LIBS

Herbal medicine (HM) now makes up a significant part of the global health care system in herbs, herbal materials, preparations, and finished products [38,39]. Due to the current trend of ‘return to nature’, HM attracts the public’s interest worldwide. HM’s safety and efficacy evaluation data are not yet adequate for meeting the criteria set by modern regulatory authorities; thus, HM has yet to be entirely accepted in certain western countries [40,41]. Research on phytochemicals and pharmacology has shown that all plants are herbaceous and can be composed of chemical components numbering in the thousands. Several targets and pathways can be exploited to exert pharmacological effects [42]. This has increased the demand for its products, expanding its markets significantly [43]. A variety of factors contribute to the quality of HM, including the botanical species, parts (leaves, stems, roots, rhizomes, flowers, seeds, etc.), processing methods (washing, drying, steaming, etc.), storage conditions (temperature, humidity, etc.), harvest time, plant years (terrestrial, soil, climate), and the cultivation environment (terrain, soil, weather, and sunlight) [39,43,44,45]. As a result of these variables, the chemistry of HM varied from one batch to another, with considerable variation in its pharmacological activities [44]. According to existing regulations and pharmacopeias, analyzing quality qualitatively and quantitatively for HM is typically conducted according to sensory information, macro-and micro-scales examinations, and quantitative analysis of a few intrinsic markers [46]. However, the sensory inspections primarily depended on personal experience and lacked a sound basis in evidence, which caused subjective differences in the qualitative assessment [47,48]. For authenticity evaluations of HM, selecting a single marker compound ignored the synergistic effect of multiple components. As a result, its effectiveness was not represented holistically [49,50]. There are techniques for evaluating the overall quality of HM by employing a non-targeted approach, a procedure known as the ‘chemical fingerprint’. Regulatory agencies, such as the WHO, CFDA, FDA, EMA, and the MFDS, accept the concept of a fingerprint for evaluating HM quality indicators [41,51,52,53]. Several analyses performed using different analytical techniques resulted in highly variable fingerprint profiles, which were not only convenient for chemical information extraction from the original dataset; moreover, it proved to be extremely challenging or the chemical information was inaccessible through general univariate analysis [54]. Chemometrics methods are increasingly used to analyze chemical fingerprints in modern quality research of HM (as a result of the development of computer science) [55]. Chemometric methods can solve multiple problems with the comparative study, investigative learning, and grouping algorithms in various fields. Various variable algorithms could be calibrated to analyze quantitatively and explore the relationships between independent and dependent variables. Chemometric analyses of data about fingerprints can provide informed judgment and understanding of mathematical modeling of chemical systems, which could pave the way for developing a thorough evaluation of HM quality.

### 6.1. Traditional Chinese Medicine (TCM)

The development of the LIBS method for elemental analysis in herbs has the potential to revolutionize the pharmaceutical industry. Detecting and identifying authentic and false components within herbs are important tasks. Due to the improvement in living standards, consumer demand is increasing for high-quality natural medicines. The temptation of economic gains has led some merchants to substitute low-quality herbs in expensive areas for high-quality herbs in cheaper areas to protect the legitimate interests of consumers and end unfair competition of this kind. Thus, it is extremely important to ensure the authenticity and safety of herbs by establishing their authenticity. LIBS and machine learning provide an efficient method to analyze TCM rapidly.

**Figure 3:**
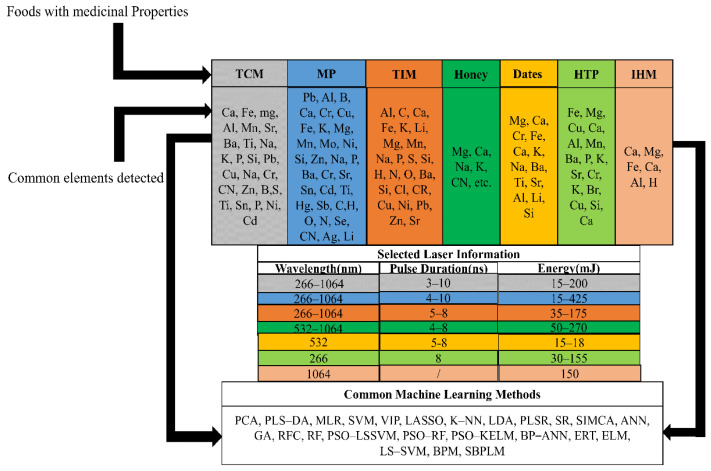
Different foods with medicinal properties and common elements detected by LIBS.

Traditional Chinese Medicine (TCM) plays an important role [56,57]. TCM is studied collaboratively in the medical and government sectors worldwide [58]. Pharmaceutical research may consider beneficial components (for example, the presence of heavy metals) and adulteration to be adverse effects (e.g., an excess concentration of heavy metals) [59,60,61]. According to TCM, pharmacodynamics is closely related to the substances absorbed and to the compounds contained within [62]. As long as heavy metal concentrations remain high in the atmosphere, TCM is in danger [63]. The popularity of TCM has also created significant challenges in identifying its products and certifying them due to issues of impurity [64]. The scientific community has also prepared special reports on the pollution caused by fake Chinese medicine and heavy metals to emphasize the importance of TCM components [62]. Researchers have made some progress in applying LIBS to study the chemistry of herbs and their biological properties in the past few years, as demonstrated by Wang et al. [65] where artemisia annua was analyzed using DP-LIBS. Measurements of the spectral intensities and signal to background ratios of magnesium (Mg) (II) 279.54 nm, Ca (II) 393.37 nm, and iron (II) 404.27 nm were made to determine the optical emission characteristics of A. annua as a result of DP-LIBS. Comparing DP-LIBS to single pulse (SP) LIBS, DP-LIBS showed higher sensitivity and precision. The enhanced DP-LIBS factors for Mg, copernicium (CN), calcium (Ca), and iron (Fe) were 7.2, 8.9, 13.8, and 3.4, respectively. This study provides valuable information regarding A. annua. Liu et al. [52] applied (LIBS) technology and chemometric techniques to determine the origin of kudzu powder. Several models that discriminate based upon a complete spectrum have been developed. Included are (ELM), (SIMCA), and (KNN), and their accuracy has consistently exceeded 99%. In terms of accuracy, KNN and RF models performed the best. Both calibrated and predicted sets of kudzu powder from different production areas demonstrated 100% accuracy. The characteristic wavelengths have been determined using principal component analysis (PCA) loadings. Both the predictive set and calibration set of discrimination models, in which characteristic wavelengths are used, have high accuracy levels of greater than 98.0%. Based on this study, LIBS may be an effective tool for identifying the authenticity of Chinese medicine. Additionally, Liu et al. [53] studied Blumea balsamifera DC using LIBS for quick elemental and provenance analysis. The multivariate nature of the LIBS data was exploited by taking advantage of (PCA) and (PLS-DA). Computed principal components of different spectral data were visually illustrated by scores and loadings of the corresponding elements. PLS-DA achieved an excellent classification result. Compared to models that use specific spectrums as input variables, the PLS-DA model produced similar discrimination performance using complete spectrum data. Specific attention was given to the down-selection of spectral lines associated with the significant elements in B. balsamifera samples. According to the outcomes, LIBS could provide rapid element analysis and provenance analysis of B. balsamifera, etc., as shown in Table 1. An example is depicted in Figure 3.

### 6.2. Medicinal Plant and Indian Herbal Medicine

Researchers are increasingly interested in the elemental studies of medicinal herbs to detect different active bio-ingredients in addition to their concentration by using different analytical techniques to assess their medicinal properties. Numerous analytical methods have been applied for measuring major and trace elements in medicinal plants, such as atomic absorption spectrometry (AAS), energy dispersive X-ray fluorescence (EDXRF), wavelength dispersive X-ray fluorescence (WDXRF), laser-induced breakdown spectroscopy (LIBS), inductively coupled plasma optical emission spectrometry (ICP-OES), particle-induced X-ray emission (PIXE), etc. Using LIBS, the spatial distribution of elements in plants and biomaterials can be determined. A high-powered laser interacts with a sample material to produce atomic and ionic emission lines along with the molecular species of the plasma. The LIBS technology offers unique advantages, such as the capability to analyze data fast, the ability to detect multi-elemental chemistry and the use of non-destructive techniques, as well as the ability to analyze solid, liquid, and gas samples in minimal time. In plant science, it has been identified as a potential “superstar” technology for the analysis of green plants [58].

Plants have been used as a source of medicine and human nourishment in India since ancient times [68,78]. In India, more than 2500 plant species possess medicinal properties. According to the WHO, it is estimated that 20,000 different species of plants are used for developing various medicines around the globe [63,79], and these studies [56,57,58,80,81,82,83,84,85,86,87,88,89,90,91,92,93,94,95,96,97] were shown in Table 2. Tripathi et al. [85] determined the distribution of micro and macroelements in four *Ocimum* species: *Ocimum basilicum*, *Ocimum sanctum*, *Ocimum gratissimum*, and *Ocimum americanum*. This was achieved by using (LIBS) and (ICAP-AES). Different Ocimum leaves were studied using LIBS spectroscopy in the 200–900 nm range, which revealed atomic lines, such as potassium (K), sodium (Na), Ca, Mg, and silicon (Si), in addition to lighter elements, such as carbon (C), hydrogen (H), oxygen (O) and nitrogen (N). ICAP-AES verified the presence and distribution of the elements mentioned previously (except the lighter elements due to their limitations). Using both techniques, Ca was the most abundant element in all species, followed by potassium (K), Mg, and sodium (Na). In addition, it was found that *O. sanctum*, in terms of mineral content, is followed by *O. basilicacum*, *O. gratissimum*, and *O. americanum*. PCA was also performed on the LIBS spectra of several *Ocimum* species, revealing that PC1 (72%) and PC2 (26%) explained 100% of the variance in the dataset. The PCA plots showed a clear separation of wild and cultivated species. A conclusion was reached that LIBS was a suitable technique because it is easy, rapid, and friendly, and compares mineral availability among *Ocimum* species. Zhu et al. [57] used (LIBS-LIF) to measure the amount of lead (Pb) in rhododendron leaves. Rhododendron leaves contain a wide range of medicinal properties. Detecting lead rapidly in rhododendron leaves is vital to monitoring the safety of drugs. Sample preparation was carried out using the powder and solid, liquid, solid transformation (SLST) methods. As a result, an increase in the 4005.78 nm signal of Pb I has been observed. The LoD values for samples A, B, and C were 0.054 mg/kg, 0.059 mg/kg, and 0.062 mg/kg, respectively, with R^2^ values of 0.997, 0.996, and 0.997, respectively, using the SLST technique, which had greater sensitivity and accuracy than the powder method. In both methods, the root mean square error of cross validation (RMSECV) values ranged from 0.53 to 2.117 mg/kg, with 1.5 to 2.8 mg/kg of lead detected by LIBS-LIF in the three samples. ICP-OES has been used to validate the results of the lead analysis. Rhododendron leaves were determined with this method for the first time in a rapid, precise, and reliable manner. Iqbal et al. [59] applied (CF-LIBS) technique was to quantitative and qualitative analyses of the sage sample. Using a rapid repetition rate of 10 Hz and pulse duration of 5 ns, a Q-switch Nd: YAG laser is configured to focus the second harmonic (532 nm) to produce the sage plasma. Five high-resolution spectrometers covering a wavelength range of 200 nm to 720 nm were used to record the emission spectra. According to the optical emission spectra of the sage sample, Fe, Ca, titanium (Ti), cobalt (Co), manganese (Mn), nickel (Ni), and chromium (Cr) are detected. Using the Boltzmann plot and the Stark-broadening line profile method, the plasma temperature and electron number density of the neutral spectral lines of the pertinent elements were determined with average values of 8855 ± 885 K and 3.89 ± 1016 cm^−3^, respectively. The elements detected in the sample were quantified using the average values of plasma parameters. The calibration-free method demonstrated that Fe constitutes 48.1% of the sample, whereas the remaining elements include Ca, Ti, Co, Mn, Ni, and Cr, each with a percentage concentration of 0.7, 5.3, 8, 11, 12.3 and 14.6%. It demonstrates the feasibility of using LIBS for analyzing the composition of major and trace elements present in plant samples and its potential for future medical applications.

### 6.3. Honey

Honey is widely used in food products and is consumed in a regular diet. Honey, however, also has therapeutic properties. A variety of sugar syrups have adulterated it due to increased demand for the product [98]. In addition, honey has limited nutritional value due to reducing effective ingredients. The contaminants can cause health problems for humans. Honey has been contaminated with numerous substances worldwide [60,61,62]. Table 3 shows several studies on detecting honey adulteration [61,64,98,99,100,101], including Lastra-Mejías et al. [102], who exposed LIBS to honey to evaluate fraud and botanical classification. The study analyzed the underlying information of adulterated samples using algorithmic methods based on chaotic parameters, such as shifted (lag-k) autocorrelation coefficients. As these algorithms can identify slight changes in the composition of honey, it has proved possible to detect these types of adulterations with a success rate greater than 90% when samples from the honey of different botanical origins are combined into one model, and over 95% when individual honey types are analyzed. Peng et al. [61] honey adulteration was measured using LIBS and chemometrics. Two common types of impurity, namely mixing acacia honey with high fructose corn syrup (HFCS) and rape honey, were quantified by univariate analysis and partial least squares regression (PLSR). Feature selection and univariate analysis of variable importance were also tested (GA), (VIP), (SR). The relationship between emission spectra from Mg II 279.58, 280.30 nm, Mg I 285.25 nm, Ca II 393.37, 396.89 nm, Ca I 422.70 nm, Na I 589.03, 589.64 nm, and K I 766.57, 769.97 nm and adulterant content was significant. The root-mean-square error (RMSE) for the SR-PLSR, VIP-PLSR, and VIP-PLSR models were 8.9, 8.2 and 4.8% for HFCS 55, HFCS 90, and rape honey, respectively. A fast and straightforward valuable method in detecting adulterated honey was identified during the study. Furthermore, Zhao et al. [99] investigated the geographical origins of acacia honey and multi-floral honey. The LIBS emissions of Ca, Na, and K were significantly affected by the source of these elements. PCA was applied to describe honey clusters from different geographical locations. Furthermore, (SVM) and (LDA) were employed to quantify the origin classification. SVM outperformed LDA, and multi-floral honey discriminant results exceeded acacia honey. The accuracy of multi-floral honey and the mean average precision were 99.7%. Based on the findings of this study, this study provided a quick method for determining geographical origins, which may be helpful for honey traceability. Multiple elements, both organic and inorganic, can be determined using LIBS simultaneously [12]. As a tool for detecting adulterants in honey, LIBS is an attractive option since it is fast, inexpensive, waste-free, and does not require sampling. Despite LIBS’s numerous applications, the technology is little explored in food analysis [10,103,104] especially for viscous products, such as honey. Since the chemical profile of sweetener syrups differs from the honey in several aspects, such as mineral content, sugars, protein, enzymes, etc. [105], the presence of impurities should influence the honey spectral profile. Moreover, many studies have demonstrated LIBS’s potential for determining physicochemical parameters or class discrimination through the correlation with the fingerprint profile of samples [106,107].

### 6.4. Date Fruits

When compared to all other food sources, dates stand out significantly. They contain almost all the essential nutrients, including sugars, making up 44% of carbohydrates (88%), nutritional fibers (6.9%/11.5%). Vitamins, proteins (2.3/5.6%), fats (0.2/0.5%), and amino acids are all low [110]. Dates are also claimed to contain over fifteen minerals and salts; however, half of the sugar in the fruit is in the form of fructose, an excessive amount of sugar for an otherwise balanced diet [102]. As well as this, dates contain approximately 0.2 to 0.5% oil in their flesh and 7.7 to 9.7% in their seeds. Dates are eaten directly and are used to make extracts, syrups, and sweeteners [111].

Given the use of refined dates in the manufacturing industries to produce various medicines and other products, a comparison of different dates is of particular interest to a broad audience [102]. Mehder et al. [12] analyzed spectroscopically the nutritional and toxic components of several dates available in Saudi Arabia. The optical emission produced is analyzed when pulsed lasers ablate a test sample. The experiment involves Mg and Ca, nutritional elements, and Cr, a toxic part. Before the elemental characterization of date samples, a condition check was performed on the (LTE) to ensure that the LIBS analysis was accurate. Several plasma-related parameters were measured, for example, the electron temperature and density of electrons. A plasma plume was formed after the date palm sample was ablated; these parameters help interpret ionization, dissociation, and excitation phenomena. A calibrated detection limit was established by plotting the LIBS signal intensity against the concentrations of standard date samples, compared with the samples of dates from various varieties, the Ca and Mg.

Regarding concentrations, the range was 187–515 mgL^−1^ and 35–196 mgL^−1^, respectively, whereas the Cr concentrations ranged from 1.72 to 7.76 mgL^−1^. In the LIBS system, the LIBS signal strength is determined by the energy of the incident laser and the delay time. The LIBS results were also validated through standard techniques, such as ICP-MS on identical or duplicate samples to those used in the LIBS analysis. It has been found that LIBS analytical results generally agree with those obtained with ICP-MS. Zafar et al. [103] spectroscopic analysis of samples from Pakistan was conducted using the LIBS technology coupled with the LA-TOF-MS technology. An Nd: YAG laser (532 nm) was used to generate the plasma, and the optical spectrum was captured using four spectrometers. Optically thin and local thermodynamic equilibrium conditions were validated for the quantitative analysis of the laser-produced plasma. According to the optical emission spectra of the date samples, Fe, Ca, Mg, K, and Na nutritional elements were detected, and toxic elements, including Ba, Ti, Sr, Al, Li, and Si. In addition, LA-TOF-MS was used to quantify samples collected on the same date. It was found that LA-TOF-MS and (CF-LIBS) were in excellent agreement, indicating that both methods can be used to analyze multiple elements in any sample, etc., as shown in Table 3.

### 6.5. Herbal Tea Plant

An estimated 18–20 billion cups of tea are consumed daily globally, making it the second most consumed beverage after water [112], grown predominantly in Southeast Asia, particularly in China and 45 other countries. In addition to many beneficial trace elements, tea leaves contain several potentially toxic ingredients. Furthermore, tea plants may have toxic elements in addition to their growth medium, agro-inputs, nutrients, soil, and other sources, such as fertilizers, industrial activities, pollution, and pesticides [113]. Chemically, tea leaves contain K, aluminum (Al), Ti, bromine (Br), Mg, Fe, Mn, Zn, copper (Cu), phosphorous (P), strontium (Sr), Na, fluorine (F), and iodine (I). Cr, Br, cadmium (Cd), and Mn are the most toxic elements, even at low concentrations.

Several of these elements are toxic and are more likely to accumulate in food chains, whereas their potential removal rate is relatively low via excretion. Ingestion of these toxic elements above the safe permissible level may lead to cardiovascular ailments, such as hypertension, distortion of the mental and nervous systems, fatigue, neurological kidney disorder, and liver and kidney dysfunction, among others. However, tea consumption may have some health benefits, such as manganese, and an excess amount of potassium benefits people with hypertension. In contrast, other advantages include preventing Parkinson’s disease, myocardial infarctions, and coronary artery diseases (Zivkovic et al. [87]. For the first time, the LIBS (TEA) applied a laser system to analyze constituent elements in tea samples. As determined by the analysis of LIBS spectra, emission lines for the elements Fe, Mg, Cu, Ca, Al, Mn, barium (Ba), P, K, and Sr were observed. Mn and Ba were analyzed quantitatively using a standard addition method. Calibration curves with regression coefficients exceeding 0.95 were obtained. LIBS results were confirmed by ICP-OES analysis, which measures the optical emission spectra. For Ba and Mn, the estimated recoveries were 99.7 and 102.3%, respectively.

In addition to confirming the application of the proposed LIBS setup for the rapid quantitative analysis of tea, the results simplify the overall analytical procedure while ensuring the feasibility of implementing LIBS technology. Food quality and safety can be controlled using this method as an analytical tool. Gondal et al. [12] utilized a 266 nm pulsed UV laser to measure different brands of tea samples. Six tea brands were analyzed using LIBS spectrums with wavelengths between 200 nm and 900 nm, and all elements were identified on the tea samples. Several tea samples were contaminated with toxic ingredients, including Br, Cr, and minerals, including iron, Ca, potassium, and silicon. Before determining the concentration of each element, the spectral assignment was conducted. A calibration curve was drawn for quantitative analysis using standard samples within the tea matrix with known concentrations. Before the spectroscopic study of the tea samples, the temperature of electrons and electron density of the plasma were also measured. Among the elements were Ca, silicon, bromine, potassium, copper, chromium, and iron found to be between 378 and 656, 96 and 124, 1421 and 6785, 99 and 1476, 17 and 36, 2 and 11 and 92 and 130 mgL^−1^, respectively, in all tea samples. The detection limits for Ca, silicon (Si), Br, K, Cu, Cr, and Fe in tea samples were 12, 1, 11, 14, 6, 12, and 22 mgL^−1^. Standard analytical techniques, such as ICP-MS, were used to determine the concentrations of each element present in tea samples to confirm the validity of the LIBS results. Concentrations detected by LIBS are in excellent agreement with those determined by ICP-MS. For spectral analysis, such as that carried out in this study, the system could be highly applicable to testing the quality and purity of food products, pharmaceutical products, etc.

### 6.6. Indonesian Herbal Medicine

Recently, many kinds of herbal medicine (with and without brand and quality assurance) are overwhelmingly available in the local market, especially in Indonesia. As presented in Table 3. Khumaeni et al. [108] identified and analyzed elements in Indonesian herbal medicine using Nd: YAG LIBS. ASAM urat, Flu tulang and Bunga naga herbal medicine were used as the samples. An Nd: YAG laser-induced a luminous plasma on the sample surface at a reduced pressure of 1333 psi. Afterward, a spectrometer obtained a spectrum of atomic emissions from the plasma. Several atomic lines were detected, including carbon (C), Mg, Fe, Ca, Al, and hydrogen (H). Herbal medicines contain major and minor elements which are beneficial to human health. Additionally, an X-ray diffraction study was conducted. These elements have also been detected in the study, as confirmed by the results. In recent years, many kinds of herbal medicine have become available in the local market, especially in Indonesia. They can be bought with or without a brand name or quality assurance. Various micronutrients and macronutrients are contained in herbal medicine, which are beneficial for the human body, including Na, Ca, K, Mg, Fe, and Zn. There are a variety of methods that can be used to analyze herbal medicine. Flame atomic spectroscopy, atomic absorption spectroscopy (AAS) and Inductively Coupled Plasma Atomic Emission Spectroscopy (ICP-AES) have been employed for the standard detection and characterization of elements in herbal medicine. Although these techniques are effective, they have several disadvantages. AAS is restricted because it can only identify one element in a single acquisition. The other technical requirement is that the sample is prepared in liquid form during the experiment, an extremely delicate process.

In addition, both systems are expensive, affecting the data acquisition cost [108]. LIBS is an atomic spectroscopic technique useful for qualitative and quantitative analysis. It typically involves using an Nd: YAG laser to induce plasma breakdowns. A multichannel optical analyzer is then used to determine the emission spectrum of the plasma, which contains several elements from the sample. Different samples have been analyzed with this technique, including solids, liquids, and gases. While there have been some developments in the use of this technique for identifying and analyzing elements in herbal medicine in recent years, it is still very rare for Indonesian herbal medicine to implement this technique.

## 7. Conclusions and Remarks

This study summarizes the applications of LIBS in herb composition analysis. Due to many compounds of various shapes and sizes occurring at low concentrations, FMPs samples require a quite complex analysis. Moreover, accurate evaluation is necessary for many aspects of life, including health, economy, and lifestyle. As a result, new approaches to FMPs analysis are constantly being explored following technological developments. Several experiments have been conducted with various, and positive results have been found for LIBS. The LIBS methodology is a trend analysis method that provides in situ, multidimensional, and remote trends analysis. As mentioned above, LIBS is used in agricultural practice and can be applied to routine studies and research. The past few years have seen increasing chemometric analysis applications to enhance the system’s capabilities.

Additionally, CF-LIBS provides an alternative to overcome various limitations as well as to conduct calibration studies for the analysis of herbal compounds. The approach enables quantitative results to be obtained without calibration data. When there is no calibration dataset for a group of samples, CF-LIBS provides an accurate measure of elemental concentration. As with conventional methods, LIBS has certain limitations as well. When used correctly, it can be a valuable tool for evaluating its effectiveness on FMPs and a valuable addition to routine field analysis for FMPs assessment.

## Figures and Tables

**Table 1:** Summary of recent different kinds of LIBS for TCM detection.

Herb	Chemometric Technique	Laser			Best Result	Ref.
		Wavelength (nm)	Pulse Duration (ns)	Energy Used (mJ)		
Saffron	PCA	1064	10	260	/	[66]
*B. balsamifera*	PCA, PLS-DA	1064	3	90	/	[54]
Herbal medicine	PCA, ANN	1064	5.82	100	99.89%	[67]
*Artemisia annua*	/	532	6	200	/	[65]
*Artemisia annua*	/	1064	5.82	100	DP-LIBS > SP-LIBS	[52]
Kudzu powder	ELM, SIMCA, K-NN, RF	532	8	200	100%	[53]
*Mentha haplocalyx*	PCA, LS-SVM	1064	3-5	400	/	[68]
*Ligusticum wallichii*	MLR	1064	5.82	100	LOD = 15.7 µg/g	[69]
*Panax notoginseng*	PLS, SVM, Lasso, LS-SVM	532	8	200	/	[70]
*Salvia miltiorrhiza*	PCA, PSO-LSSVM, PSO-RF, PSO-KELM	1064	5.82	100	94.87%	[71]
*Salvia miltiorrhiza*	RF	532	/	110	96.19%	[72]
Nigella seeds (Kalonji)	/	532	5	200	/	[73]
*Codonopsis pilosula*	/	1064	10	/	/	[74]
*Angelica pubescens Biserrata*	ANN	1064	5.82	100	/	[75]
*Astragalus*	/	1064	8	15	/	[76]
Cinnamon	/	266	8	50	/	[77]

**Table 2:** Summary of LIBS applications for MP detection.

Herb	Chemometric Technique	Laser			Best Result	Ref.
		Wavelength (nm)	Pulse Duration (ns)	Energy Used (mJ)		
Sage (herb)	PCA, BP-ANN	1064	4	400	/	[58]
Rheum. Officinale	/	1064	10	15	R^2^ = 0.996	[80]
Species of herbs	/	1064	8	100	/	[81]
*Allium cepa* (Onion)	/	532	/	/	/	[82]
Medicinal plant leaves	BPM	1064	8	200	7 torr	[83]
Antimalarial herbal plants	SVM, LDA, K-NN	445	/	/	SVM = 100%, KNN = 100%	[84]
*Moringa Oleifera* seed	/	266	8	30	/	[85]
*Moringa Oleifera*	/	266	8	30	/	[85]
Miracle Moringa tree leaves	/	266	8	50	/	[86]
*Moringa Oleifera*	/	/	/	175	/	[87]
*Thymus Daenensis*	Cluster analysis	200–1100	/	/	/	[88]
*Ficus religiosa*	/	/	/	/	/	[89]
Poaceae Species	/	532	5	/	/	[90]
Root tissues of vicia faba	/	266, 1064	/	5, 100	/	[91]
Turmeric	/	532	4	425	/	[92]
Rhatany root	/	/	8	50	/	[93]
*Zanthoxylum Armatum*	/	532, 1064	5	200, 400	/	[94]
Rhododendron leaves	/	1064	6	/	R^2^ = 99.7%	[57]
Medicinal plant samples	PLS-DA	1064	10	17	/	[95]
Ocimum species	PCA	532	4	425	/	[56]
Mixtures of herbal medicines	PCA	1064	10	17	/	[96]
Mint (pudina)	BPM, SBLPM	532	5	/	/	[97]

**Table 3:** Summary of LIBS applications for Honey, Dates fruit, IHM, TIM and HTP detection.

Herb	Chemometric Technique	Laser			Best Result	Ref.
		Wavelength (nm)	Pulse Duration (ns)	Energy Used (mJ)		
Honey	Algorithm based on chaotic parameters	/	6	270	>90%	[64]
Honey	PLS, PLS-DA	1064	8	50	100%	[98]
Honey	LDA, ERT	1064	4	70	>90%	[99]
Honey	LDA, RFC	1064	5	70	>90%	[100]
Honey	PCA, SVM, LDA	532	/	30	99.7%	[101]
Honey	PLSR, GA, VIP, SR	532	/	80	RMSE = 8.9%	[61]
Dates	/	/	8	15–18	/	[11]
Dates	/	532	5	/	/	[102]
Indonesian herbal medicine	/	1064	/	150	/	[108]
Rhizomes of black turmeric	/	266	8	35	/	[104]
Fresh henna leaves	/	532	5	/	16.0 ± 0.2 mg/Kg	[105]
Emblica Officinalis seeds	/	/	/	175	47.09% (*p* < 0.001)	[106]
Shilajit	/	1064	/	100	/	[107]
Peppermint tea	/	/	/	155	99.7%	[109]
Tea samples	/	266	8	30	/	[10]

## Data Availability

The data presented in this study are available on request from the corresponding author.

## Abbreviations

LIBS	Laser-Induced Breakdown Spectroscopy
ML	Machine Learning
HM	Herbal Medicine
FMPs	Foods with Medicinal Properties
TCM	Traditional Chinese Medicine
MP	Medicinal Plants
TIM	Traditional Indian Medicine
IHM	Indonesian Herbal Medicine
HTP	Herbal Tea Plant
LTE	Local Thermal Equilibrium
WHO	World Health Organization
CFDA	China Food and Drug Administration
FDA	Food and Drug Administration
EMA	European Medicines Agency
MFDS	Ministry of Food and Drug Safety
LF	Laser-induced fluorescence
PCA	Principal Component Analysis
SVM	Support Vector Machine
PLS	Partial Least Squares
PLS-DA	Partial Least Squares Discriminant Analysis
ANN	Artificial Neural Network
BP-ANN	Back Propagation Artificial Neural Network
ELM	Extreme Learning Machine
SIMCA	Soft Independent Modeling of Class Analogy;
K-NN	K-Nearest Neighbor
RF	Random Forest
MLR	Multiple Linear Regression
LS-SVM	Least Squares Support Vector Machines
LASSO	Least Absolute Shrinkage and Selection Operator
PSO-LSSVM	Particle Swarm Optimization-Least Squares Support Vector Machine
PSO-RF	Particle Swarm Optimization-Random Forest
PSO-KELM	Particle Swarm Optimization-Kernel Extreme Learning Machine
CF-LIBS	Calibration Free-LIBS
SLST	Solid liquid solid transformation
LDA	Linear Discriminant Analysis
GA	Genetic Algorithm
VIP	Variable Importance In Projection
LTE	Local Thermodynamic Equilibrium
SR	Selectivity Ratio
MSL	Mars Science Laboratory
LIF	Laser-induced fluorescence
ERT	Extremely Randomized Trees
ICP-OES	Inductively Coupled Plasma-Optical Emission Spectrometry
RFC	Random Forest Classifiers
HPLC-RID	High-Pressure Liquid Chromatography-Refractive Index Detector
EO	Electro-optical; BPM, Boltzmann Plot Method
SBLPM	Stark Broadened Line Profile Method
DP-LIBS	Double Pulse Laser-Induced Breakdown Spectroscopy
SP-LIBS	Single Pulse Laser-Induced Breakdown Spectroscopy
PAT	Process Analytical Technology
